# The exploration of factors related to treatment retention in Narcotics Anonymous members: a qualitative study

**DOI:** 10.1186/s13011-019-0205-6

**Published:** 2019-04-11

**Authors:** Rostam Jalali, Asie Moradi, Fateme Dehghan, Samira Merzai, Mostafa Alikhani

**Affiliations:** 1 Department of Nursing, School of Nursing and Midwifery, Kermanshah University of Medical Sciences, Kermanshah, Iran; 2 Department of Psychology, Faculty of Social Sciences, Razi University, Kermanshah, Iran; 3 Substance use Prevention Research Center, Health Institute, Kermanshah University of Medical Sciences, Kermanshah, Iran

**Keywords:** Substance use disorder, Narcotic anonymous, Treatment retention

## Abstract

**Background:**

Elimination of psychological dependence to substance is more difficult than elimination of physical dependence, and needs to time, going through several stages, and internal care. The aim of this study was to exploring the factors related to treatment retention in Narcotics Anonymous members.

**Methods:**

In a qualitative study and by individual interview, 12 recovered participants were interviewed. The participants were substance user, whom recovered for more than two years. The data were gathered by purposeful sampling and through recording and transcribing interviews. Data analysis were done by qualitative content analysis through three steps: conceptualization, interview and data analysis.

**Results:**

After analyzing data, two main categories had emerged: “personal-psychological” and “social” factors. Personal-psychological" factors includes: self-knowledge, change of attitude, self-confidence, consistency in treatment, living in the moment and “social” factors include interaction with others, group of sympathizers, reformation of social and familial relationships, reclaiming the social position, supports received from others, and supports received from the generalized network.

**Conclusion:**

Recovered individuals are in need to emotional supports and reclaiming their positions in the family and society play an important role in their treatment retention. Keeping the Substance users away from drugs is not the basic step of substance use treatment, but the necessary supports and special cares should be given to the Substance users after elimination of physical attachment, so that the psychological dependence can be eliminated as well.

## Background

Substance abuse disorders have typically been defined as either symptom-based or diagnosis-based. Symptom-based conceptualizations focus on the types and severity of problems related to the use of a particular substance, while diagnosis-based descriptions are based on whether or not a person meets a specified set of criteria generally associated with the abuse of a particular substance [1]. Substance abuse disorders are a major contributor to the economic and healthcare burden in Australia; costing $55 billion annually through crime, road accidents, workplace productivity losses and healthcare costs [2] and abuse of tobacco, alcohol, and illicit drugs is costly to USA, exacting more than $740 billion annually in costs related to crime, lost work productivity and health care [3] Substance abuse is a lifestyle accompanied by physical, mental and spiritual suffering for the substance abusers, their families and society [4]. There has been increasing awareness that for many individuals substance use can be a chronic disorder, characterized by multiple relapses and additional treatment episodes over time [5]. Substance use is a primary mental health problem in the Iranian population. Studies have estimated that approximately 1.12 (2.1%) million Iranians aged 15–64 years old meet the Substance use disorder according to the DSM-5 criteria [6]. The most recent NSDUH survey estimated that 22.6 million persons met criteria for a Substance use disorder. Of these, 3.2 million were classified with dependence on or abuse of both alcohol and illicit drugs, 3.8 million were dependent on or abused illicit drugs but not alcohol, and 15.6 million were dependent on or abused alcohol but not illicit drugs. These estimates have remained relatively stable since 2002 [7]. The most visible manifestation of the opioid epidemic is the rising number of overdose deaths, estimated at more than 33,000 in 2015 [8]. Substance use is a complex illness. It is characterized by intense and, at times, uncontrollable drug craving, along with compulsive drug seeking and use that persist even in the face of devastating consequences [9]. Substance use is a leading cause of preventable morbidity and mortality among youth; indeed, more cases of death, disease, and disability are caused by Substance use than by any other preventable health condition [10].

Treatment for Substance use disorders range from medical to psychosocial, flexible to structured and from outpatient options to intensive inpatient programs [11]. Despite promising advances in biopsychosocial treatments, Substance use disorders continue to significantly impact the health of people worldwide, due in part to the treatment gap between optimal care and currently available services [12]. when treatment is delivered to clients seeking services for Substance use problems, alcohol and drug use decreases, engagement in crime is reduced, and other social functioning measures improve during and following treatment [1]. A study reported a positive relationship between length of time spent in treatment and favorable outcomes, a finding that spans treatment modalities, programs, and treatment models [13]. At the same time, many clients do not remain in Substance use treatment long enough to reap its benefits. Although the percentage of clients who do not complete Substance use treatment due to dropout or expulsion varies widely and can be difficult to measure because treatment modalities have diverse treatment expectations, some general trends have been observed. Lower estimates of the dropout rates for inpatient alcohol and drug treatment programs are around 20%, while upper estimates can reach 70% [1]. The studies conducted in mental health centers in various countries found dropout rates routinely fluctuate between 35 and 55% [14, 15]. According to the studies conducted by the Moos, although Substance use -focused self-help groups uses a variety of theories [16] but based on other studies 12-Steps Narcotics Anonymous (NA) couldn’t resolve treatment retention properly [17, 18].

In light of these observations, treatment retention has emerged as an important intermediate outcome measure in the study of Substance use treatment. The increased utilization of research methodologies, assessment procedures, and statistical analyses designed to evaluate the inherent complexities of treatment processes (i.e., engagement, participation, therapeutic relationship) and how they relate to treatment retention and outcomes is allowing researchers to expand areas of inquiry and to continue building the theoretical and applied knowledge base in the treatment for Substance use disorders. In light of these demanding circumstances, questions arise about how Substance users percept recovery and what factors can affect on treatment retention. Diverse methodological techniques have been employed across various programs serving assorted clients to investigate the relationships amongst client, program, and treatment attributes, treatment retention, and eventual outcomes [12, 17–19].

Unfortunately, no consistent “treatment dropout” profile has been detected, and the generalizability of these findings are often questioned at the local level because of the stark differences that exist between particular treatment programs and their clientele and those studied [20]. From a clinical perspective, it is difficult for a program to examine treatment outcomes without first learning about who is entering treatment and who is staying in treatment. There is a critical need for knowledge about the process of substance use recovery, about the challenges, about useful resources as well as about the positive outcomes of recovery. Such knowledge can provide recovering persons, their families and service professionals with realistic expectations for recovery outcomes, knowledge about the timeframes within which such outcomes are likely to be achieved, and the strategies and processes through which they are facilitated [21].

The identification of variables that positively and negatively relate to treatment retention will further assist in the creation of an assessment procedure that allows clinicians to quickly and efficiently detect clients who may be at risk for dropout. Ultimately, such knowledge can begin to inform the design of interventions aimed at enhancing treatment retention, which can potentially improve treatment outcomes as the positive relationship between retention and outcomes is well-established in the literature. Additionally, certain combinations of variables may relate to whether or not a client completes treatment, thus retention enhancing interventions should target multiple areas to address the inherent complexity of the presenting problems of clients engaging in Substance use treatment. Hence, the aim of this study was to exploring the factors related to treatment retention in Substance users.

## Methods

### Design and sample

In a qualitative study and by content analysis 12 participants were interviewed. Participants were selected through purposeful sampling and continued until data saturation. Saturation is used in qualitative research as a criterion for discontinuing data collection and/or analysis [22]. Participants were Substance users referred to Substance use treatment centers and camps, and also Narcotics Anonymous which had been treated at least 2 years ago. Before doing the study, NA experienced and talkative members who interested to participate and completed an informed written consent were selected. After coordination for date and place of interview, semi-structured interview were done in a friendly and calm atmosphere. The interview started with general questions such as “What has caused you to have a successful treatment? And what factors have caused you not to be drawn to using substance once again?” and continued with more detailed and follow up questions based on the participants’ answers. Interviews duration were from 35 to 60 min. Interviews were recorded and then transcribed. The study settings were Substance use treatment centers and camps, and also Narcotics Anonymous associations in Kermanshah city (west of Iran).

### Analysis

The recorded sessions were transcribed verbatim immediately after each session. We analyzed data according to the approach proposed by Graneheim and Lundman [23]. Which involved the following steps: The transcribed interviews were regarded as units of analysis. Meaning units were extracted from the interview texts after several reviews and achieving a good understanding of the statements. Then the meaning units were shortened into condenses meaning units. A number of codes, also referred to as the ‘labels’ of the condensed meaning units, were then formed. The codes were then assessed in terms of similarities in their content and sub-categories and categories were developed respectively. The meaning units and codes were extracted and were then classified and extracted into themes. To clarify the extracted codes, categories and themes, direct quotations are provided from the interviews in the results section.

### Rigor

We attempted to achieve qualitative rigor using methods suggested by Lincoln and Guba [24]. These methods consists of prolonged engagement with subjects, peer check, external check, presenting details on the participants as well as the study setting, providing a background on the subject and discussing any relevant literature, explaining the process of recording the raw data and providing a lengthy explanation of the processes of coding and analysis.

## Result

All of the 12 participants were men, among them, 10 were married and 2 were single (Table 1).

**Table 1:** Demographic characteristic of participants

No	Age	Education level	Job	Substance abuse duration	Substance abuse type	Cessation duration	Center of cessation
1	42	Bachelor science	Employee	11	Smoking Cannabis Alcoholic Heroin Injection	12	Injury reduction institute DIC
2	43	Diploma	Freelance	7	Opium	14	NA
3	46	Diploma	Freelance	10	Opium Heroin	16	NA Detoxification Therapeutic agents
4	35	Elementary	Freelance	10	Smoking Cannabis Alcoholic Heroin Injection Opium Crack	7	NA
5	29	Elementary	Freelance	5	Smoking Cannabis Alcoholic Heroin Injection Opium	7	NA
6	36	Primary	Freelance	12	Opium	4	Cessation center
7	49	Primary	Freelance	22	Opium Heroin	5	Therapeutic community
8	48	Diploma	Freelance	18	Opium	10	Cessation center
9	37	Elementary	Worker	10	Opium	5	Cessation center
10	34	Diploma	Freelance	12	Heroin Injection Opium	4	Cessation center
11	28	Elementary	Worker	12	Opium Alcoholic	2	Cessation center
12	36	Elementary	Worker	17	Opium	3	Individually

**Fig. 1:**
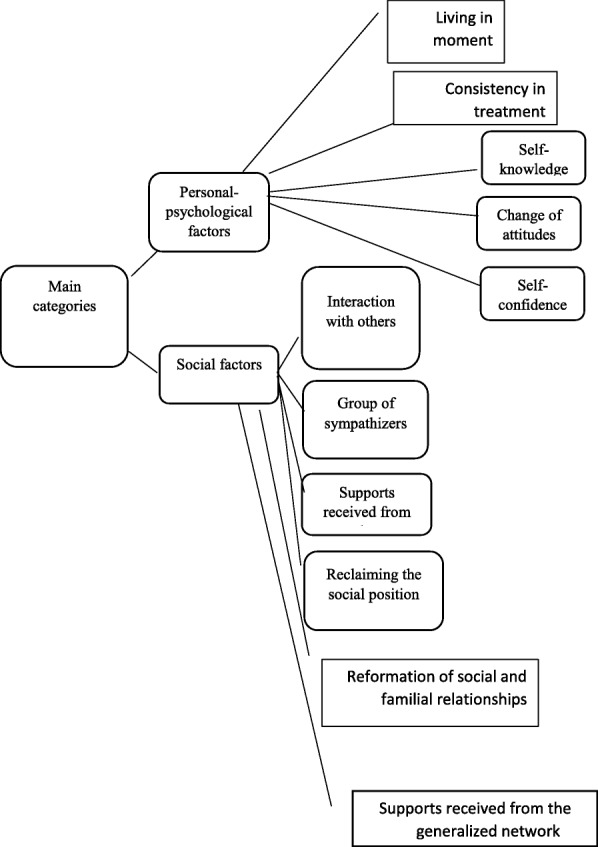
Main categories and subcategories

After extracting the contents of interviews and categorizing in various levels, eventually two main categories “personal-psychological” and “social” factors were extracted. Figure 1 shows categories and subcategories.

### Personal-psychological factors

Changes that are created in the persons and causes them to recovering, is generally related to the changes in their attitudes and beliefs. These changes include: gaining back the confidence that had been decreased during Substance use period, changes in attitude towards life and outside world and specially Substance use, having insight and self-awareness and knowledge about personal abilities and even weaknesses. Based on participants experiences, (facilitator factors include self-knowledge, change of attitude, self-confidence, consistency in treatment, living in the moment).

#### Self-knowledge

As said by the interviewee, they had achieved a more complete self-knowledge, and claimed this self-awareness to be a result of attending NA meetings.

Participant no. 2 expressed: “I got persistent in the treatment when I discovered my role in the past and present happenings, and did not blame other people for my substance use anymore”. Also, another participant (participant no.10) stated: “After joining NA groups, I discovered that I’m also an ill person and many of other ill people like me have referred to here and have been treated, and this awareness towards my illness did not give me the negative feeling of being substance user”.

#### Change in attitude

New beliefs and change in attitudes, beliefs and opinions which exist in substance use period causes treatment retention, and though this is not easily achieved, it is not impossible either. Participant no. 1 expressed: “A higher and more powerful force was created in me, in which I believed, and I could stay persistent by trusting it”. Participant no. 8 also said: “I changed my mind and believed that I should never try using drugs for once”. Also participant no. 2 talked about change in his viewpoint and thoughts: “I’ve made positive changes in myself and I’m not the same person as before”. This change in viewpoints and beliefs is effective and significant in treatment retention to the extent that participant no. 2 said: “I changed my personality and identity after recovering”. Participant no. 6 also said: “I haven’t fought with anyone after Recovering, and I’m not willing to lose the dignity and respect that I’ve achieved in the society”. Based on statements of participant no. 3: “I believe that my family, friends and even the society need me to be treated”. Participant no. 10 expressed: “This is my perception that not only drugs do not solve any problems, but also they add to my problems”. As is observed, these people have created new definitions for their lives, and even made changes in their beliefs and identities.

#### Self-confidence

Lack of self-confidence is one of the problems that is observed clearly in those having the tendency to use drugs. Creating self-confidence is followed by self-knowledge, and these two are necessary to each other. Self-confidence helps the individuals to believe in treatment and retention in substance use treatment. Participant no. 3 said: “I felt worthy for myself and society after Recovering”. Participant no. 5 stated: “I obtained the ability to say no to drugs”.

#### Treatment retention

Improving the lifestyle of the patient is important for the continuation of Substance use treatment. Joining to the NA association meetings every week, even after a few consecutive years with retention, is a clear sign of this category. Participant No. 12 stated: “I will not be persistent on Recovering tomorrow unless I adhere to a set of principles,” as Participant No. 1 said: “After 12 years of Recovering, I’m participating in ceremonies special to Recovering and recovering communities. I’m announcing that I’m a 12 year’s old treated addict”.

#### 3Living in just now

Living at moment or just now means that, with all our senses, we are alert and aware of the present. It means not thinking about the past or living in it, nor it is anxious or worried about the future. Participant No. 3 said: “Do not go to the past and regret, nor worry about future.”

### Social factors

Based on the results obtained from the current study, the quality and type of communication with others and the supports they received in their social interaction with others were considered to be of the most important factors in treatment retention.

#### Interaction with others

The quality and type of interactions existing in social communication network as “interacting with others” that three subcategories are identifiable in relation to it as follows: “group of sympathizers, reformation of social and familial relationships and retrieving the social position”.

#### Group of Sympathizers

The participants considered one of the causes of their treatment retention to be attending the NA meetings and being close to people that have had the same conditions as them, people that perceive the same pain and understand what has happened to them. They believe that NA members know what kind of help and support the newbies need, without needing to ask them. Each newbie has a guide which has been one of the treated people himself. The environment of NA and seeing people that have had the same conditions as me and are now recovered, intensified the belief that I can be clear as well. Participant no. 9 stated: “I consulted with the professionals and I have abandoned my selfishness, pride and self-orientation”. Participant no. 1 also said: “A guide who had the history success treatment himself support me, and was accessible to me whenever I needed him, to the extent that I called him in the midnights when I was not feeling good and he talked to me”. Participant no. 5 reiterated: “I understood that here are the people who are similar to me, and all of them have been using drugs”.

#### Reformation of social and familial relationships

Based on the personal experience of participants, the NA members are supposed to pass some stages in the treatment process, one of which is that the substance user should reciprocate the damages he has done to others, and this in turn prepares the ground for the reformation of their relationships with others. First, the relationship of the substance user is broken with the users and traffickers, and other relationships are reformed. The participants talked about the improvement of their relationships with family, relatives, friends and neighbors. Even some of them emphasized that they have also improved their relationship with themselves and their God. Reformation and improvement of relationship prepared them for treatment retention. For example, participant no. 4 expressed: “My behavior towards others has changed and I don’t fight with anyone and I’m not the same person as before”. Participant no. 7 also said:“ before Recovering, I was disrespected by my children and family, but after treatment, they respect me again because my behavior towards them has changed”.

#### Reclaiming the social position

Substance user which have lost their social positions during the substance use period, try to retrieve it gradually in the process of treatment. Reclaiming the social position strengthens them in treatment retention. The participants explained that the society has accepted them somehow, and has given them some responsibilities that shows their trust. Participant no. 12 expressed: “I am now in such social position that I’ve tried so hard to achieve, and I’m not willing to lose it at any price”. Participant no. 2 said: “After the substance use treatment, because of the trust and credit I had gained from being a member of NA, I got several licenses to establish damage reduction centers, and even I designed various models to successful treatment”. Participant no. 1 also said: “After my successful treatment, I founded an substance use treatment center and got a job through it, and in my opinion, one of the most important factors of treatment retention is having a job”.

#### Supports received from others

In the current research, the participants mentioned two types of support during treatment period. First, the supports given by family, relatives, friends and colleagues (linked supports) and second, the supports given by NA association (generalized supports).

Linked relationship networks supports the individual to stay in the treatment by encouraging them and creating an intimate relationship with them. Of course, there are emotional supports provided for the individual by NA in the treatment period, but the linked relationship network is emotionally more important for the individual, and this emotional force will have more effect if coming from linked relationship networks. Participant no. 1 stated: “My family supported me while I was going through the treatment. For example, my mom cooked my favorite dish in the number of NA members and brought it there, and this event was so heartwarming to me”. This participant said: “Remarriage and acceptance and support of my wife was one of the main reasons for my treatment retention”. Participant no. 3 also said: “My family supported me economically and spiritually, during treatment period and after that”.

The participants said that when their relationship with their families improves and they are accepted by the family, returning to the normal conditions and consistency on treatment will have more persistence. This trust and acceptance in the work and living environment is important and valuable for under-treatment person. Participant no. 11 stated: “After Recovering, my family respect me so much and consult with me about important matters, and they truly accept me”.

#### Supports received from the generalized network

Most of participants were NA members, and the special supports given to them by the association have enabled them to be treatment retention. The NA members help each other to know about substance use, and the older ones enlighten the path for others to stay persistent in treatment. The NA helps substance users overcome their problem with their abilities, by knowing them. Participant no. 5 expressed: “In the beginning when I joined NA, I was recommended to take a guide”. Participant no. 1 claimed: “In my opinion, one of the most effective ways of treatment is becoming a member of NA”. Participant no. 3 also stated: “The sense of belonging to NA has caused me to be at the service of new members as a treated person, and working in the heart of substance use has resulted in me being more therapeutically persistent”. Participant no. 10 stated: “I’m still using the 12 steps taught to me in the NA, and they were not only useful in my treatment, but also they are now still my way up in the life”.

Support and the awareness that the NA gives the recovering people in the form of 12 steps, have been very effective in treatment retention, and is the lost piece of puzzle that its place was obviously empty in the substance use period and repetitive, unfruitful treatments. This matter is also confirmed by the experience of the participants. Participant no. 12 said: “I had reached my end, until God took mercy on me and I was introduced to the NA by a treated friend, and there, I got aware that I was ill and what matters is my illness, not my substance use. This awareness was the first of the 12 steps of the association, then other steps were shown to me which I’m still using as a useful, important means after 7 years of being clear. I have tried so hard to reach the position I have now and I haven’t achieved them in one day, also another step of the association was that I have not become ill in one day, so I won’t get treated in one day, and because of this, persistence in treatment and complete avoidance is necessary in treatment retention”.

## Discussion

The results of the current study showed that after getting treated in NA association, these were the most effective factors of treatment retention: being a member of NA, accepting oneself as an ill person, and receiving support from the sympathizers (other treated members of NA), which led to a change in the individuals’ attitude and self-knowledge. Also, reformation of social and familial relationships, receiving emotional and financial supports from family members, and also increasing the individual’s self-confidence and reclaiming his social position and getting a job are the other effective factors of treatment retention in NA members.

In recent years, various researchers have studied the matter of prevention and treatment of Substance use from different medical, psychological, social and spiritual aspects, and have suggested several approaches to resolve the problem. In this regard, many centers and associations with the purpose of preventing and treating Substance use have been founded in recent years, and some of these centers have had good achievements in improving the conditions of the substance users individual. The result of our study gained from interaction with NA association members. This interaction support and encourage recovering persons and cause to overcome on their problems. Other studies supported our finding. In a study they find that five factor relating to perceptions of successful recovery: Improved understanding of their substance use, reduced Substance use, improved physical and psychological health, relationship success and employment success cause attending to a cessation program [25]. In another study they find that a designated program with group interaction can help to stress management, refusal skills, pros of drug use, and drug use resistance self-efficacy, excluding cons of drug use [26]. and also Ali found inequalities in access to opioid antagonist therapy can be an obstacle for recovered persons [27]. One may name NA network as one of the most successful for treatment retention, which has been able to attract many people and help them improve by using universal experiences in this field. Previous studies have also confirmed the success of self-help group of NA members in treatment and success treatment retention, improvement of life quality and reinforcement of the wills of the substance users who are undergoing the treatment [21, 22].

In explanation of why the substance users tend to get treated in a group, based on numerous studies, there is a positive and significant correlation between unsuccessful individual recovering and tendency to joining NA associations. This confirms the past history of a human being and their success or failure in the past affects their current behavior. In fact, based on finding, because most of the substance user decide to quit by themselves and fail to be persistent in their Recovering, they gain the tendency to get treatment in the NA associations. The powerful and positive relationship between social support of NA members and successful Recovering with tendency to remain a member of this group is a confirmation of the theories that claim that human being is a social creature, and he needs to gain the respect and interest of others, belong to a relationship network and have mutual commitments. Based on this, the support received from the similar group mates in special group of NA associations instills the sense of being valuable to the Recovering person, which is very effective in successful treatment of this group. In other words, it can be said that this finding confirms the satisfaction of NA members of being a member of this association [28]. The positive effect of being a NA member on retention treatment is in line with theory of “social identity” that based on which people make meaning from their self-image by becoming a member of a group, and define their identity with this sense of belonging. Based on this theory, the substance user persons gain positive social identity by creating a positive in-group distinction, and this identity helps them to attend the meetings continuously, which leads to a successful treatment. Thereupon, recovered substance use in a group is the most successful way to be treatment retention, because of the social supports that the individual receives from other members of the group, and also because of the identification which is created for him inside the group [29, 30]. The theorists of social capital believe that persons gain a capital in their social interactions, which is effective in their flow of life later. Social capital includes social communication networks and trust, which are destroyed by addiction, but strength and weakness of this change is proportionate with the environment and social communication network that the person had belonged to before the addiction. Some researchers have looked at the role of social factors in not returning to substance use. In a study, a researcher tried to understand how individual or community members change their daily behaviors, and how substance users after years of substance use quitted and returned to normal life. The results showed some conditions caused formation of substance use, as well as the conditions contribute to the quitting [31].

Some studies have also examined the effect of membership in the group on treatment retention [32–34]. Among these studies, some have focused on the impact of residence in community-based centers. For example, studying Akochakian et al. On 30 substance user before and after therapeutic community showed that membership in this group could be an effective treatment program for improving the mental status of substance user and reducing their willingness to return [35].

The results of the current study showed that increasing self-confidence and reclaiming the social position of the under-treatment person is one of the effective factors of treatment retention. Previous studies support this finding [36, 37]. Therefore, it can be said that being a member of associations and groups can prepare the ground for increasing of the self-confidence of the under-treatment persons and be effective in their treatment retention of substance use.

The participants of current study emphasized that the instruction they have received in NA associations in the form of 12 steps, have had a great effect on changing their viewpoint towards themselves and their lives. Thus, the NA members receive the necessary supports and instructions from the association, which can be effective in eliminating their psychological dependence even after Recovering. The results of previous researches also emphasize on the role of the NGO in prevention of substance use and preventive approaches with social orientation [38].

### Limitations

The current study faced some limitations: One of them was that the participants were mostly the members of NA, and people who have used personal methods or methods other than NA’s and achieved success, were not studied.

## Conclusion

Recovered individuals are in need of kind but not pitying behavior of those surrounding him more than any other time, and receiving financial and emotional supports and reclaiming his position in the family and society play an important role in his treatment retention. It should be considered that the model of the preventive medical plan that consists of preventive and therapeutic plans for treatment of substance use, is not effective by itself. Plus, merely keeping the Substance users away from drugs is not the basic step of substance use treatment, but the necessary supports and special cares should be given to the Substance users after elimination of physical attachment, so that the psychological dependence can be eliminated as well.

## Abbreviations

NA: Narcotic Anonymous
NGO: Non-Governmental Organization
